# The inhibitory effects of polypyrrole on the biofilm formation of *Streptococcus mutans*

**DOI:** 10.1371/journal.pone.0225584

**Published:** 2019-11-27

**Authors:** Hidenobu Senpuku, Elif Bahar Tuna, Ryo Nagasawa, Ryoma Nakao, Makoto Ohnishi

**Affiliations:** 1 Department of Bacteriology I, National Institute of Infectious Diseases, Shinjuku-ku, Tokyo, Japan; 2 Department of Pediatric Dentistry, Faculty of Dentistry, Istanbul University, Istanbul, Turky; 3 Graduate School of Life and Environmental Sciences, University of Tsukuba, Tsukuba, Ibaraki, Japan; LSU Health Sciences Center School of Dentistry, UNITED STATES

## Abstract

*Streptococcus mutans* primary thrives on the biofilm formation on the tooth surface in sticky biofilms and under certain conditions can lead to carious lesions on the tooth surface. To search for a new preventive material for oral biofilm-associated diseases, including dental caries, we investigated the effects of polypyrrole, which contains an electrochemical polymer and causes protonation and incorporation of anion under low pH condition, on the biofilm formation of *S*. *mutans* and other streptococci. In this study, polypyrrole was applied in biofilm formation assays with the *S*. *mutans* strains UA159 and its *gtfB* and *gtfC* double mutant (*gtfBC* mutant), *S*. *sanguinis*, *S*. *mitis* and *S*. *gordonii* on human saliva and bovine serum albumin-coated 96-well microtiter plates in tryptic soy broth supplemented with 0.25% sucrose. The effects of polypyrrole on biofilm formation were quantitatively and qualitatively observed. High concentrations of polypyrrole significantly inhibited the biofilm formation of *S*. *mutans* UA159 and *S*. *sanguinis*. As an inhibition mechanism, polypyrrole attached to the surface of bacterial cells, increased chains and aggregates, and incorporated proteins involving GTF-I and GTF-SI produced by *S*. *mutans*. In contrast, the biofilm formation of *gtfBC* mutant, *S*. *sanguinis*, *S*. *mitis* and *S*. *gordonii* was temporarily induced by the addition of low polypyrrole concentrations on human saliva-coated plate but not on the uncoated and bovine serum albumin-coated plates. Moreover, biofilm formation depended on live cells and, likewise, specific interaction between cells and binding components in saliva. However, these biofilms were easily removed by increased frequency of water washing. In this regard, the physical and electrochemical properties in polypyrrole worked effectively in the removal of streptococci biofilms. Polypyrrole may have the potential to alter the development of biofilms associated with dental diseases.

## Introduction

*Streptococcus mutans* primarily thrives on the tooth surface in sticky biofilms that are formed in extreme aciduric and acidogenic environments and consist of up to 700 different species of microorganisms in oral cavities [1–6]. The sticky biofilms formed by *S*. *mutans* are principally produced by insoluble glucan formation induced by the principal enzymes GTF-I and GTF-SI in conditions supplemented with an optimal concentration of sucrose [7, 8]. *S*. *mutans* is an adherent bacteria and is one of the primary pathogens in the development of dental caries [7, 8]. *S*. *mutans* produces acids and is itself highly tolerant to acid; it also produces bacteriocin, possesses high-affinity systems for the assimilation of many carbohydrate sources, such as glucan and fructan, and forms sticky biofilms [9, 10]. Biofilms are constructed by an extracellular matrix composed of exopolysaccharides (EPSs), lipids, proteins, and eDNA [11–13]. eDNA is one of the major components in biofilms and is released naturally or by cell death and lysis of bacteria [14–16]. Cell death facilitates bacterial adherence, aggregation, accumulation and increasing biofilm biomass through the release of eDNA into the extracellular matrix [13, 17]. The degradation of eDNA by the addition of DNase I results in a significant decrease in biofilm formation [18, 19]. eDNA has important functions as an attachment factor for surfaces and an adhesive factor among bacteria during the initial stage of biofilm formation [11, 20].

Polypyrrole (see S1 Fig) is an organic conductive polymer formed from a pyrrole ring structure [21, 22]. Polypyrrole materials exhibit high electric conductivity, which is moderate in the air, and have deionization properties, thermostability, and a favorable electrochemical nature. It is formed easily, chemically and electrochemically. In addition, polypyrrole is not toxic and has a positive charge [23–25]. Particularly, the availability of electronic positive holes increases so that polypyrrole is positively charged with electricity, and the coplanarity between the chains provides a favorable condition for increased conductive ability [23, 26]. These attractive properties are important for the production of biosensors for controlled drug release systems [28], proteins [29–32] and DNA [33, 34] by chemical or electrochemical means in aqueous media for synthesis and relatively long-term stability [23, 24, 27]. In biomedical use, polypyrrole is usually and electrochemically generated with the incorporation of anionic species containing negatively charged biological macromolecules such as proteins and polysaccharides to provide composite material [35].

To search for a new preventive material to oral biofilm-associated diseases including dental caries, we investigated the effects of polypyrrole on the biofilm formation of *S*. *mutans* and other streptococci. Higher concentrations of polypyrrole significantly inhibited the biofilm formation of *S*. *mutans* and *S*. *sanguinis*, and in contrast, lower concentrations of polypyrrole induced biofilm formation in streptococci that do not produce sticky polysaccharides. However, the induced biofilm was easily removed by repeated washes. In this study, the role of the structure of polypyrrole was explored in biofilm formation with and without insoluble glucan. The usage of polypyrrole for oral hygiene might be promising for the prevention of biofilms-associated oral diseases such as dental caries.

## Materials and methods

### Bacterial strains and culture conditions

The *S*. *mutans* laboratory strains UA159, MT8148 and GS-5; *S*. *mutans* clinical isolates FSM-5, FSC-7 and FSC-8 [36]; *Streptococcus sobrinus* strains OMZ176 and AHT; *Streptococcus sanguinis* strain ATCC 10556, *Streptococcus gordonii* strain ATCC 10558, *Streptococcus mitis* strain ATCC 6249, and *S*. *mutans* UA159 *gtfB* and *gtfC* double mutant (*gtfBC* mutant) [37] were maintained and grown in brain heart infusion broth (BHI, Becton/Dickinson, Sparks, MD) at 37°C with 5% CO_2_ aerobic atmosphere (Gas pack: Mitsubishi Gas Chemical Co., Inc. Tokyo, Japan). Tryptic soy broth without dextrose (TSB, 286220, Becton/Dickinson, Sparks, MD) supplemented with 0.25% sucrose was used for biofilm formation, growth curves, aggregation assays, and the visual observation of biofilm cells.

### Human saliva collection

Whole saliva samples were collected from 3 healthy human volunteers (24–28 years old) after stimulation by chewing paraffin gum and pooled into ice-chilled sterile bottles over a period of 5 min. The samples were clarified by centrifugation at 10,000 x *g* for 10 min at 4°C, sterilized using a 0.22-μm Millex-GP Filter Unit (Merck Millipore, Darmstadt, Germany), and coated onto wells in plates (Sumitomo Bakelite, Tokyo, Japan) for biofilm formation assays.

### Ethics statement

This study was approved by the ethics committee of the National Institute of Infectious Diseases for the usage of human saliva in coating plates (IRB approval number: 397). Prior to enrolment, written informed consent was obtained from each subject based on the code of ethics of the World Medical Association (declaration of Helsinki).

### Biofilm formation

Biofilms formation was developed in 96-well polystyrene microtiter plates (Sumitomo Bakelite, Tokyo, Japan), which were previously coated with human saliva, BSA, anti-PAc serum [38], anti-PAc monoclonal antibody [39] and anti-GbpC serum [40] for 1h at 4°C. After pre-coating, wells were washed with phosphate buffered saline (PBS) 2 times. Biofilm formation assays were performed using a previously described procedure [36]. Wells containing 180 μl of TSB with 0.25% sucrose were inoculated with 20 μl of *S*. *mutans* UA159 and *gtfBC* mutant, *S*. *sanguinis*, *S*. *mitis* and *S*. *gordonii* from a cell culture with an optical density (OD_600_) of 0.4. The plates were then incubated with various concentrations of polypyrrole (Sigma-Aldrich, Co., St. Louis, MO) or hyaluronic acid (MW 5,000–150,000, Tokyo Chemical Industry, Co. LTD, Tokyo, Japan) at 37°C with 5% CO_2_ for 16 h. To observe the effects of hyaluronic acid on polypyrrole-dependent biofilm formation, polypyrrole was pretreated with 7 mg/ml hyaluronic acid for 1 h at 37°C and applied to the biofilm formation assay. After incubation, the planktonic cells were removed by washing with distilled water (DW), and the adherent cells were stained with 0.25% safranin for 15 min. After washing with DW 2 times, safranin was extracted from biofilm with 70% (v/v) ethanol, and quantitatively measured by the absorbance at 492 nm.

### Observation of bacterial growth

Overnight bacterial cell cultures grown in BHI were diluted 1:60 in fresh TSB with 0.25% glucose at pH 7.0. *S*. *mutans* UA159 were grown with and without polypyrrole at 37°C in a 5% CO_2_ atmosphere for 16 h. To observe morphology of grown cells, bacterial cells on glass slides were observed using Gram staining and a phase-contrast microscope (×400). To clearly observe effects of polypyrrole on the growth of *S*. *sanguinis* ATCC 10556 and the *S*. *mutans* strains (UA159, MT8148, GS-5, FSC-8, FSM-5 and FSC-7), cells samples after incubation for 16 h in TSB with 0.25% sucrose and BHI supplemented with various concentrations of polypyrrole were poured on BHI agar plates. The cell colony numbers were counted on BHI agar plates after 48 h incubation and effects of polypyrrole on the cell growth were assessed.

### SDS-PAGE

Before SDS-PAGE, culture supernatants (experiment 1 and 2) from *S*. *mutans* UA159 were cultivated with and without 2.5 mg/ml polypyrrole for 1 h at 37°C. Prior to electrophoretic analysis, the control, which contained PBS mixed with 2.5 mg/ml polypyrrole, and the culture supernatants with and without 2.5 mg/ml polypyrrole were diluted with an equal volume of SDS-PAGE sample buffer {0.06 M Tris-HCl (Amersham Pharmacia Biotech, Buckinghamshire, UK), pH 6.8; 20% glycerol (Wako Pure Chemical Industries Ltd, Osaka, Japan); 1% (vt/vol) SDS (Wako); 1% 2-mercaptoethanol (2-ME, Sigma-Aldrich); and 0.0012% bromophenol blue (Wako)}. The SDS-PAGE samples were heated at 100°C for 3 min prior to loading on the gel. The samples were then run on a 12.5% polyacrylamide gel (e-PAGEL, ATTO Corp., Tokyo, Japan) in the presence of 0.025 M Tris-HCl, 192 mM glycine (Wako) and 0.1% (wt/vol) SDS. Electrophoretic separation of the proteins was carried out for 70 min at 40 mA. Gels were stained with Coomassie blue to observe the proteins.

### Western blotting

After SDS-PAGE, proteins were transferred onto Immobilon PVDF membranes (Millipore, Bedford, MA) and blocked with 2.5% skim milk in TBST (50 mM Tris, 2.7 mM KCl, 0.138 M NaCl, 0.05% Tween 20, pH 7.6) for 1 h at room temperature. The membranes were probed with anti-GTF antisera [41] from rabbits diluted 1:500 overnight at 4°C or with the control nonimmunized antibody (Wako) diluted 1:15,000 in 1.25% skim milk/TBST for 1 h at room temperature. HRP-labeled anti-goat secondary antibody (Merck kGaA) was used to detect the antibodies. Optical emission signals on the proteins were produced by enhanced chemiluminescence (ECL Western Blotting Substrate, Thermo Scientific, Southfield, MI) and detected by exposing the X-ray film (FUJI FILM, Kanagawa, Japan) for different times.

### Observation of live/dead cells in biofilm formation

Biofilms were stained using the FilmTracer Live/Dead Biofilm Viability kit (Molecular Probes, Inc., Eugene, OR), and the reagents were applied to the biofilms at final concentrations of 5 μM SYTO9 and 30 μM propidium iodide. Biofilms were incubated with the dyes at room temperature for 20–40 min before being imaged by a confocal microscope, LSM700 Meta NLO CLSM (Carl Zeiss Inc, Thornwood, NY). Confocal images for biofilm formation were visualized by the analysis software ZEN (Carl Zeiss).

### Measurement of insoluble polysaccharide

*S*. *mutans* UA159 suspensions were prepared by the same procedure as the biofilm formation assay. Two hundred microliters of cell suspension was mixed with 1800 μl of TSB supplemented with 0.25% sucrose. The mixture was added with 0.5% polypyrrole to the wells of 6-well microtiter plates (Corning Incorporated, NY). After inoculation, the plates were incubated at 37ºC for 4, 8, 12 and 16 h in an aerobic atmosphere of 5% CO_2_. The plates were rinsed with PBS 3 times, and the biofilms on the wells were removed by a sterilized scraper. The removed biofilm samples were collected by centrifugation at 10,000 × *g* for 10 min, and the pellet was suspended in dH_2_O. The biofilm-associated insoluble glucan was extracted as described by Freedman *et al*. with partial modifications [42]. The samples were treated with 1 N NaOH at 37ºC for 2 h. Then, the cells were removed by centrifugation at 10,000 × *g* for 10 min. The insoluble polysaccharides in the supernatants were precipitated by 75% (v/v) ethanol at -20ºC for 2 h. The precipitate was washed 3 times with 70% ethanol by centrifugation at 10,000 x *g* for 30 min at 4ºC and dissolved in 1 N NaOH. The carbohydrate content was determined by the phenol-sulfuric acid method.

### Statistical analyses

The growth turbidity, biofilm biomass and initial attachment data were expressed as the means ± standard deviations (SD). In the biofilm assay, the statistically significant differences between the bacteria with and without polypyrrole or no polypyrrole and various concentrations of polypyrrole were determined using the unpaired *t*-test and one-way ANOVA with Bonferroni correction (IBM SPSS statistics 24, Chicago, IL), respectively. The independent experiment in triplicate was performed 3 times. The data were presented as the mean ± SD of triplicate experiments and compared biofilm formation levels with polypyrrole with levels with no polypyrrole. *P-*values less than 0.05 were considered statistically significant.

## Results

### Inhibitory effects of polypyrrole on GTF-dependent biofilm formation

**Fig 1:**
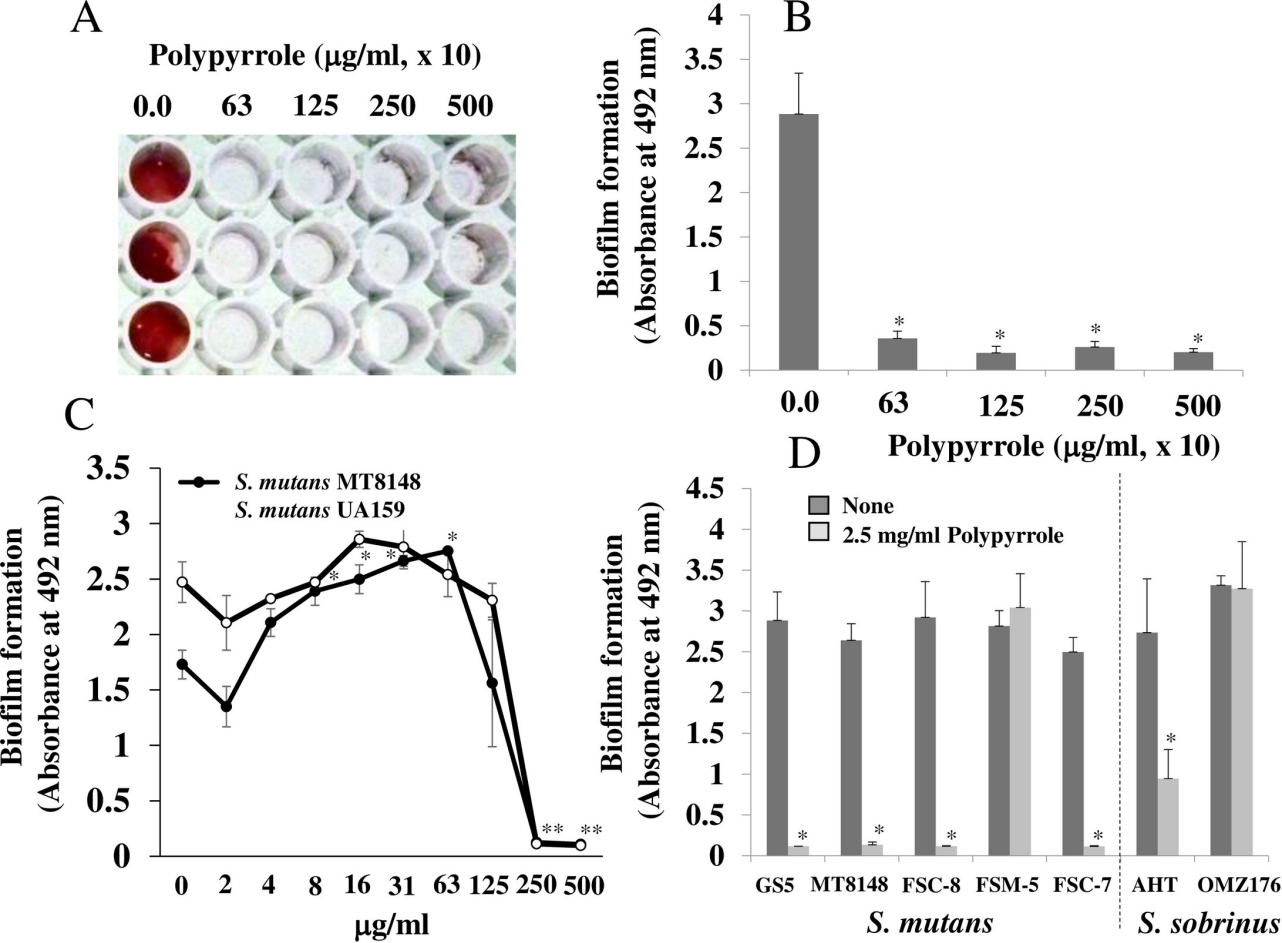
The inhibitory effects of polypyrrole on the biofilm formation of *S*. *mutans*. Effects of various concentrations of polypyrrole on the biofilm formation of *S*. *mutans* UA159 were observed on human saliva–coated 96-well microtiter plates after incubation for 16 h in TSB supplemented with 0.25% sucrose. A: Biofilm formation in *S*. *mutans* was present at high concentrations of polypyrrole (63, 125, 250 and 500 x 10 μg/ml) on human saliva–coated 96-well microtiter plates. B: Biofilm formation in *S*. *mutans* UA159 was quantitatively analyzed at high concentrations of polypyrrole. C: Biofilm formation in *S*. *mutans* UA159 and MT8148 was quantitatively analyzed at various concentrations of polypyrrole. D: Biofilm formation in the *S*. *mutans* strains GS-5 and MT8148, clinical isolates FSC-8, FSM-5, and FSC-7, and *S*. *sobrinus* AHT and OMZ176 were quantitatively analyzed at 2.5 mg/ml polypyrrole. The data indicated the mean ± SD of triplicate experiments. The independent experiments were performed 3 times, with similar results obtained in each. The asterisks indicated a significant difference between 2 groups (*: *p* < 0.05, no polypyrrole vs polypyrrole).

The inhibitory effects of polypyrrole on the insoluble glucan-dependent biofilm formation of the wild-type *S*. *mutans* laboratory strains UA159 (Fig 1A, 1B and 1C), MT8148 (Fig 1C) and GS-5 (Fig 1D) in TSB supplemented with 0.25% sucrose were observed. Higher concentrations (more than 250 μg/ml) of polypyrrole inhibited biofilm formation, but other concentrations did not. In contrast, 8–125 μg/ml polypyrrole significantly increased biofilm formation compared to the control: no polypyrrole in MT8148 (Fig 1C). The inhibitory effects of polypyrrole on *S*. *mutans* UA159 biofilms were observed in plastic tubes, and the aggregates that were stained a black color by polypyrrole likely led to the inhibition (S2 Fig). Inhibition was also observed in the *S*. *mutans* clinical isolates FSC-8 and FSC-7, and *S*. *sobrinus* AHT but not in the *S*. *mutans* clinical isolate FSM-5 and the *S*. *sobrinus* strain OMZ176 at 2.5 mg/ml (Fig 1D). Concentrations of polypyrrole (320 μg/ml-2.5 mg/ml) did not inhibit the growth of the *S*. *mutans* strains (S3 Fig). Moreover, these concentrations (40–320 μg/ml) of polypyrrole did not have cytotoxic effects on epithelial cells (Ca9-22)(S4 Fig). Therefore, 320 μg/ml of polypyrrole was at least one of appropriate concentrations that allowed inhibition to biofilm formation and safety concentration to epithelial cells.

**Fig 2:**
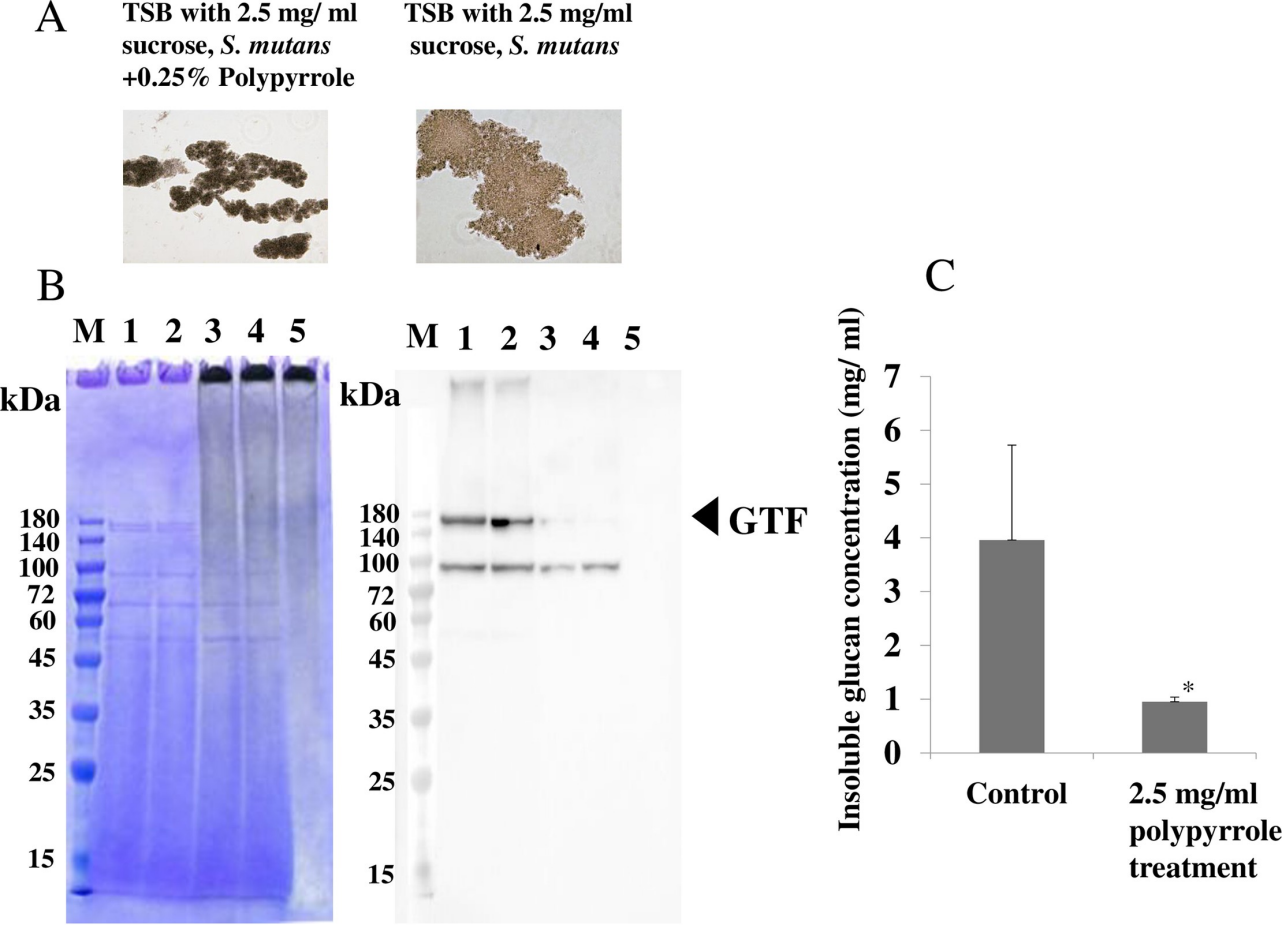
The effects of polypyrrole on the aggregation and production of insoluble glucan in *S*. *mutans*. The effects of polypyrrole on the aggregation and production of insoluble glucan by *S*. *mutans* were observed after incubation for 16 h in TSB supplemented with 0.25% sucrose. A: Aggregations of *S*. *mutans* UA159 were observed at a high concentration of polypyrrole (2.5 mg/ml). B: Effects of polypyrrole (2.5 mg/ml) on the proteins produced by *S*. *mutans* UA159 in culture supernatant were analyzed by SDS-PAGE and Coomassie Brilliant Blue staining, and western blotting. M: Molecular size marker, 1: culture supernatant experiment 1, 2: culture supernatant experiment 2, 3: culture supernatant experiment 1 + 2.5 mg/ ml polypyrrole, 4: culture supernatant experiment 2 + 2.5 mg/ ml polypyrrole, 5: 2.5 mg/ ml polypyrrole/PBS. C: The production of insoluble glucan by *S*. *mutans* UA159 was quantitatively analyzed at a high concentration of polypyrrole (2.5 mg/ml). The data indicated the mean ± SD of triplicate experiments. The independent experiments were performed 3 times, with similar results obtained in each. The asterisks indicated a statistically significant difference between 2 groups (Student’s *t*-test; p < 0.05, vs no treatment control).

To observe the mechanisms of inhibition by polypyrrole on the biofilm formation of *S*. *mutans*, a culture of *S*. *mutans* UA159 was mixed with 2.5 mg/ml polypyrrole and grown in TSB with 0.25% sucrose for 16 hours. After centrifugation, aggregated cells were observed under a microscope. The sizes of the aggregates with bacteria and polypyrrole were smaller than those without polypyrrole (Fig 2A). The aggregated cells were stained with the original black color of polypyrrole. In contrast, the plate surface was not stained with this color. As one of the mechanisms for biofilm inhibition, the effects of polypyrrole on the absorbance of the various components produced by *S*. *mutans* in culture during biofilm formation were considered. After mixing polypyrrole and the culture supernatants from *S*. *mutans*, the effect of polypyrrole on the absorbance of the various proteins in the culture supernatants was observed by SDS-PAGE. Two main protein bands, GTFs (GTF-I; 162 Da, GTF-SI; 149 kDa), disappeared by mixing the culture supernatants with 2.5 mg/ml polypyrrole, as opposed to the culture supernatants without polypyrrole (Fig 2B left). The ninety kDa protein band was also decreased by mixing the culture supernatants with polypyrrole. To confirm the presence of GTFs and the effects of polypyrrole on the proteins in the culture supernatant, western blotting using anti-GTF antibodies was performed. The GTF bands in culture supernatants from experiments 1 and 2 disappeared by mixing with polypyrrole (Fig 2B right). Therefore, it is possible that active components that play roles in biofilm development, such as GTF-I and GTF-SI, are absorbed and inactivated by polypyrrole. To clarify the inhibitory effects of polypyrrole on GTF activities, polysaccharides were quantitatively measured in the aggregated cells after mixing cultures of *S*. *mutans* with polypyrrole in TSB with 0.25% sucrose. GTFs were inactivated by polypyrrole as the polysaccharide concentration decreased with the addition of polypyrrole (Fig 2C).

### Effects of polypyrrole on the GTF-I and GTF-SI-independent biofilm formation of *S*. *mutans*

**Fig 3:**
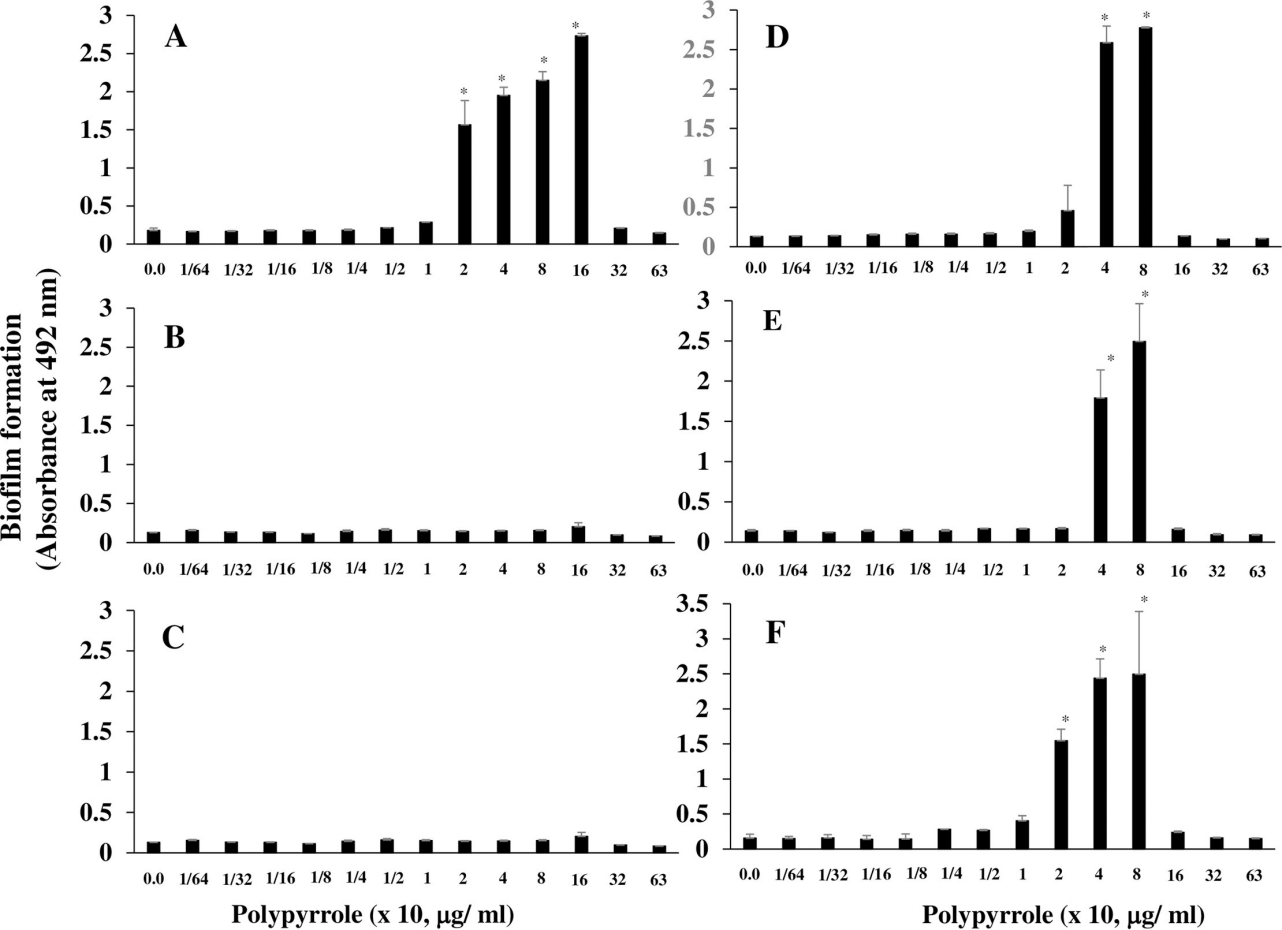
Biofilm formation in *S*. *mutans* induced by polypyrrole. Effects of polypyrrole on the biofilm formation of *S*. *mutans* were observed in the condition without glucan. Biofilm formation in *gtfBC* mutant was quantitatively assessed in conditions with various concentrations of polypyrrole on human saliva-coated (A), uncoated (B), BSA-coated (C), anti-PAc antiserum-coated (D), K4A (anti-PAc monoclonal antibody)-coated (E) and anti-GbpC antiserum-coated (F) 96-well microtiter plates in TSB supplemented with 0.25% sucrose. The data indicated the mean ± SD of triplicate experiments. The independent experiments were performed 3 times, with similar results obtained in each. The asterisks indicated a significant difference among multiple groups (ANOVA with Bonferroni correction; *p-values* < 0.05, various concentrations of polypyrrole vs no polypyrrole control).

In the condition without insoluble glucan, polypyrrole may induce different effects on the adherence and aggregation of *S*. *mutans* from those in the condition with glucan. To study the effects of polypyrrole on *S*. *mutans* cells in the condition without glucan, polypyrrole was applied in the biofilm formation assay using *gtfBC* mutant, which lacks the expression of GTF-I and GTF-SI to produce insoluble glucan. Polypyrrole induced biofilm formation of *gtfBC* mutant on human saliva-coated 96-well microtiter plates in TSB with 0.25% sucrose (Fig 3A) at a concentration of 20–160 μg/ml but not at a concentration higher than 320 μg/ml. In contrast, biofilm formation was not observed in the BSA-coated and uncoated 96-well microtiter plates at any concentration (Fig 3B and 3C). Therefore, biofilm formation may be dependent on the interaction between *S*. *mutans* and human salivary components. To observe whether specific binding interactions between *gtfBC* mutant and the salivary components on the plate surface were associated with biofilm formation, anti-PAc antiserum, a monoclonal antibody (K4A) to PAc, and anti-GbpC antiserum, which binds to cell wall-anchored proteins (PAc and GbpC) on *S*. *mutans*, were used to coat the wells instead of human saliva. All antibodies induced biofilm formation at the same concentration (40–80 μg/ml) of polypyrrole that induced biofilm formation as the human saliva coating (Fig 3A, 3D, 3E and 3F). The interaction and specific binding between the salivary components and the *S*. *mutans* surface proteins were important for biofilm formation induced by polypyrrole. However, the biofilms that formed were easily removed by increased washes with DW. Therefore, the biofilm induced by polypyrrole on the solid surface was weak.

### Observation of the inhibitory effects of polypyrrole on biofilm formation

**Fig 4:**
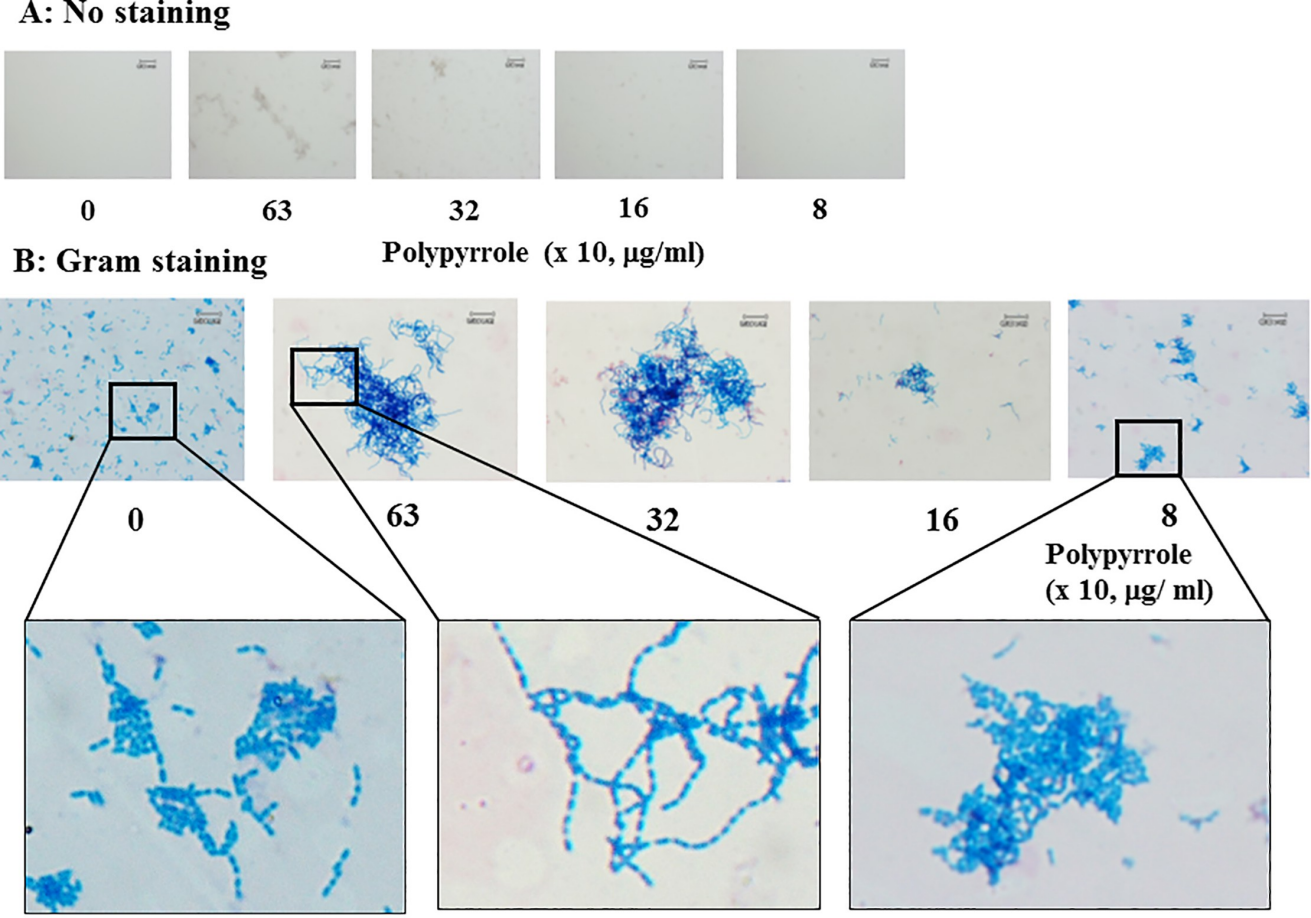
Microscopic observation of biofilms treated with high concentrations of polypyrrole. *GtfBC* mutant was incubated with 0 (control), 8, 16, 32 and 63 x 10 μg/ml polypyrrole and stained without (A) or with (B) a Gram staining kit. The aggregates were observed by microscopy. Squares were presented as an enlarged view. Representative data from more than three independent experiments were presented in the images.

Forty–80 μg/ml polypyrrole induced biofilm formation, but higher concentrations (more than 320 μg/ml) of polypyrrole did not induce biofilm formation (Fig 3A, 3D, 3E and 3F). To clarify why higher concentrations of polypyrrole did not induce biofilm formation, the *gtfBC* mutant was incubated with higher concentrations of polypyrrole, visualized by Gram staining and observed by microscopy. The *gtfBC* mutant cells that were aggregated with polypyrrole were stained by the black color of polypyrrole originally observed at higher concentrations (320 and 630 μg/ml) in conditions without Gram staining (Fig 4A). This was also observed in aggregates of *S*. *mutans* and polypyrrole in a plastic tube assay (S2 Fig). These data indicated that polypyrrole physically and largely attached to *S*. *mutans* cells and induced aggregation. After Gram staining, long chains of *S*. *mutans* were observed by crystal violet at 320 and 630 μg/ml polypyrrole and were entangled and physically aggregated among themselves (Fig 4B). However, *S*. *mutans* did not form long chains in 80 μg/ml polypyrrole, which induced biofilm formation (Fig 3A). These results indicated that chains of *S*. *mutans* were elongated without dispersion by attachment of large amounts of polypyrrole on the *S*. *mutans* cells. The entangled long chains were easily removed from the plate surface by washing with DW.

### Effects of polypyrrole on the biofilm formation of early colonizers

**Fig 5:**
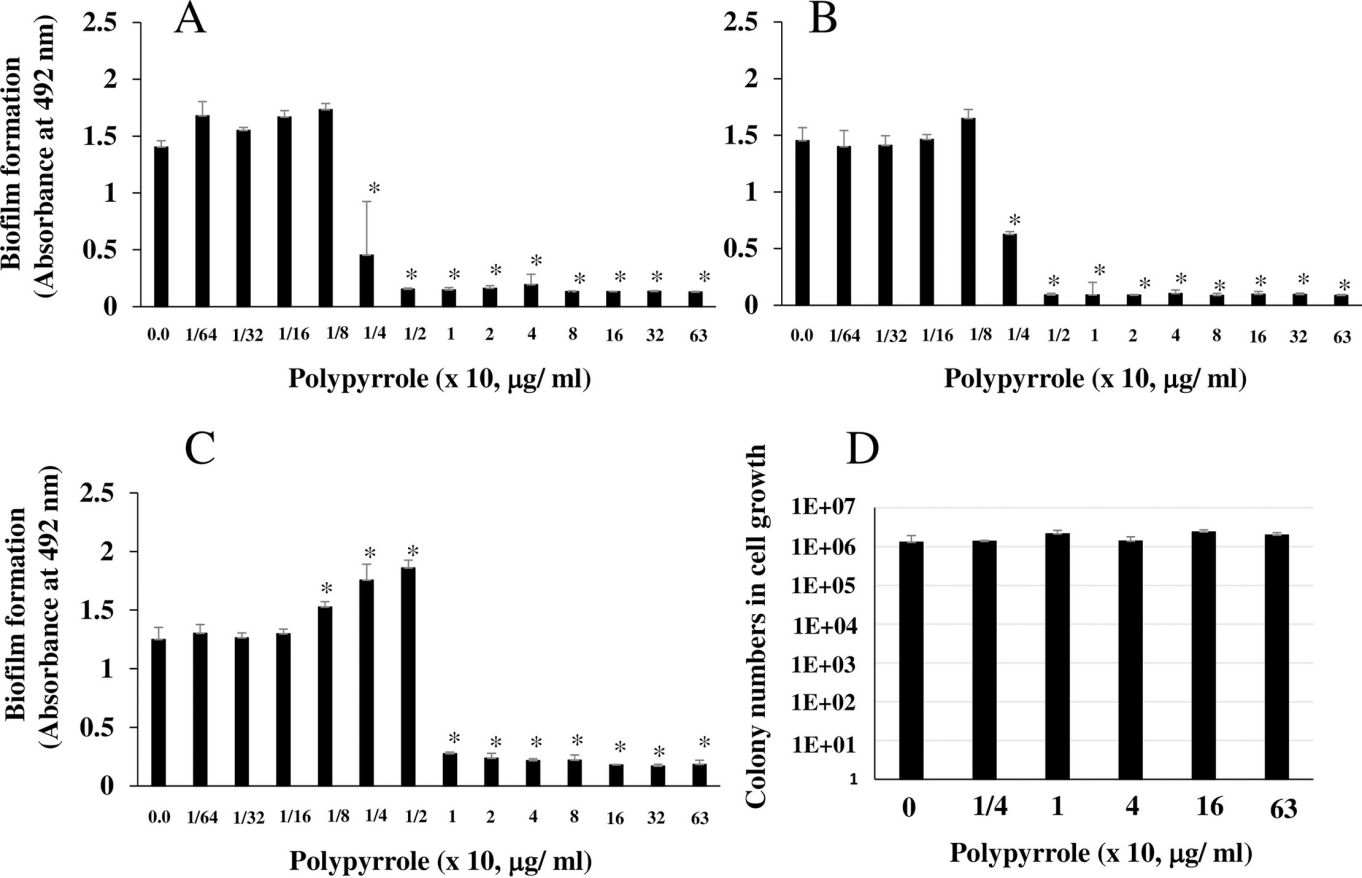
Biofilm formation by *S*. *sanguinis* induced by polypyrrole. The effects of polypyrrole on the biofilm formation of *S*. *sanguinis* were observed. Biofilm formation by *S*. *sanguinis* ATCC 10556 was quantitatively assessed with various concentrations of polypyrrole on human saliva-coated (A)-, uncoated (B), and BSA-coated (C) 96-well microtiter plates in TSB supplemented with 0.25% sucrose, including various concentrations of polypyrrole. (D) The growth of *S*. *sanguinis* was assessed by measuring the cell colonization numbers on BHI agar plates after 16 h of incubation in TSB supplemented with 0.25% sucrose, including various concentrations of polypyrrole. Small C indicated no polypyrrole as a control. The data indicated the mean ± SD of triplicate experiments. The independent experiments were performed 3 times, with similar results obtained in each. The asterisks indicated a statistically significant difference among multiple groups (ANOVA with Bonferroni correction; *p-values* < 0.05, various concentrations of polypyrrole vs no polypyrrole control).

**Fig 6:**
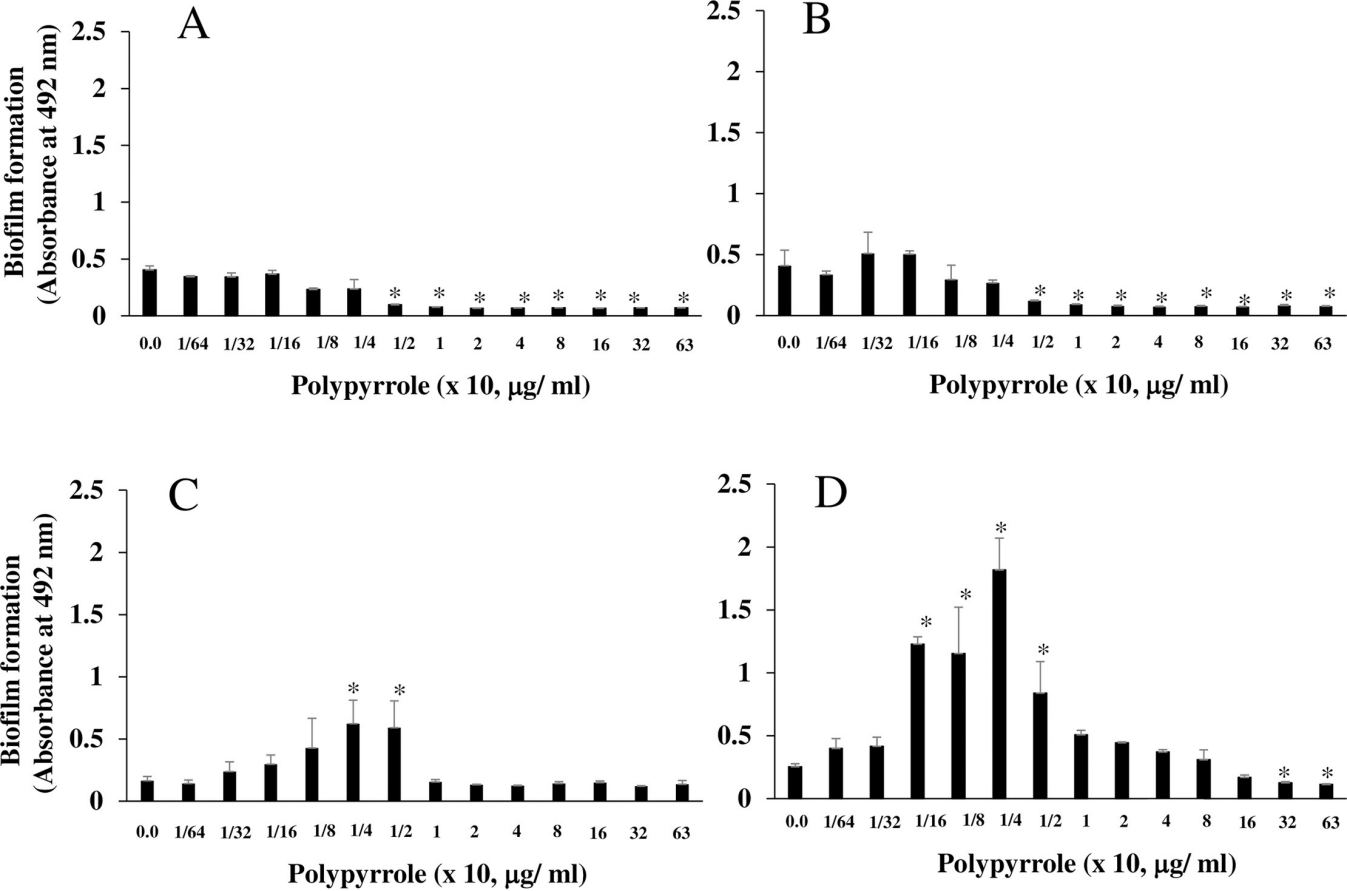
Biofilm formation by *S*. *gordonii* and *S*. *mitis* induced by polypyrrole. The effects of polypyrrole on the biofilm formation by *S*. *gordonii* and *S*. *mitis* were observed. Biofilm formation by *S*. *gordonii* ATCC 10558 (A and C) and *S*. *mitis* ATCC 6249 (B and D) was quantitatively assessed in various concentrations of polypyrrole on uncoated (A and B) and human saliva-coated (C and D) 96-well microtiter plates in TSB supplemented with 0.25% sucrose in various concentrations of polypyrrole. The data indicated the mean ± SD of triplicate experiments. The independent experiments were performed 3 times, with similar results obtained in each. The asterisks indicated a statistically significant difference among multiple groups (ANOVA with Bonferroni correction; *p-values < 0*.*05*, various concentrations of polypyrrole vs no polypyrrole control).

Certain concentrations (20–160 μg/ml) of polypyrrole weakly induced biofilms of *gtfBC* mutant (Fig 3A). To clarify the effects of polypyrrole on biofilm formation by other biofilm-forming bacteria that do not produce insoluble glucan, *S*. *sanguinis* ATCC 10556, *S*. *mitis* ATCC 6249 and *S*. *gordonii* ATCC 10558, which are early colonizers on tooth surfaces, were applied to the biofilm formation assay with various concentrations of polypyrrole in TSB supplemented with 0.25% sucrose. Increases in the biofilm formation of *S*. *sanguinis* were not observed at any concentration of polypyrrole on the uncoated and BSA-coated 96-well microtiter plates (Fig 5A and 5B). More than 1/4 x 10 μg/ml polypyrrole completely inhibited biofilm formation in all coating conditions. In contrast, on the human saliva-coated 96-well microtiter plate, 1/8-1/2 x 10 μg/ml polypyrrole increased biofilm formation compared with the control biofilm of *S*. *sanguinis* induced in the no polypyrrole control (Fig 5C). Higher concentrations of polypyrrole (2.5–630 μg/ml) did not inhibit the growth of *S*. *sanguinis* (Fig 5D). Appropriate concentrations of polypyrrole for biofilm formation by *S*. *sanguinis* were different from those by *gtfBC* mutant. For the other early colonizers, increases or decreases in the biofilm formation of *S*. *gordonii* and *S*. *mitis* were not observed at lower concentrations of polypyrrole (1/64-1/4 x 10 μg/ml) on the uncoated 96-well microtiter plates (Fig 6A and 6B). However, biofilm formation was inhibited by polypyrrole at concentrations greater than 5 μg/ml. In contrast, on the human saliva-coated 96-well microtiter plate, concentrations of 1/4-1/2 and 1/16-1/2 x 10 μg/ml polypyrrole significantly increased biofilm formation by *S*. *gordonii* and *S*. *mitis*, respectively (Fig 6C and 6D). However, the biofilm formation of all streptococci was also removed by increased frequency of water washing.

### Effects of the positive charge of polypyrrole on biofilm formation

**Fig 7:**
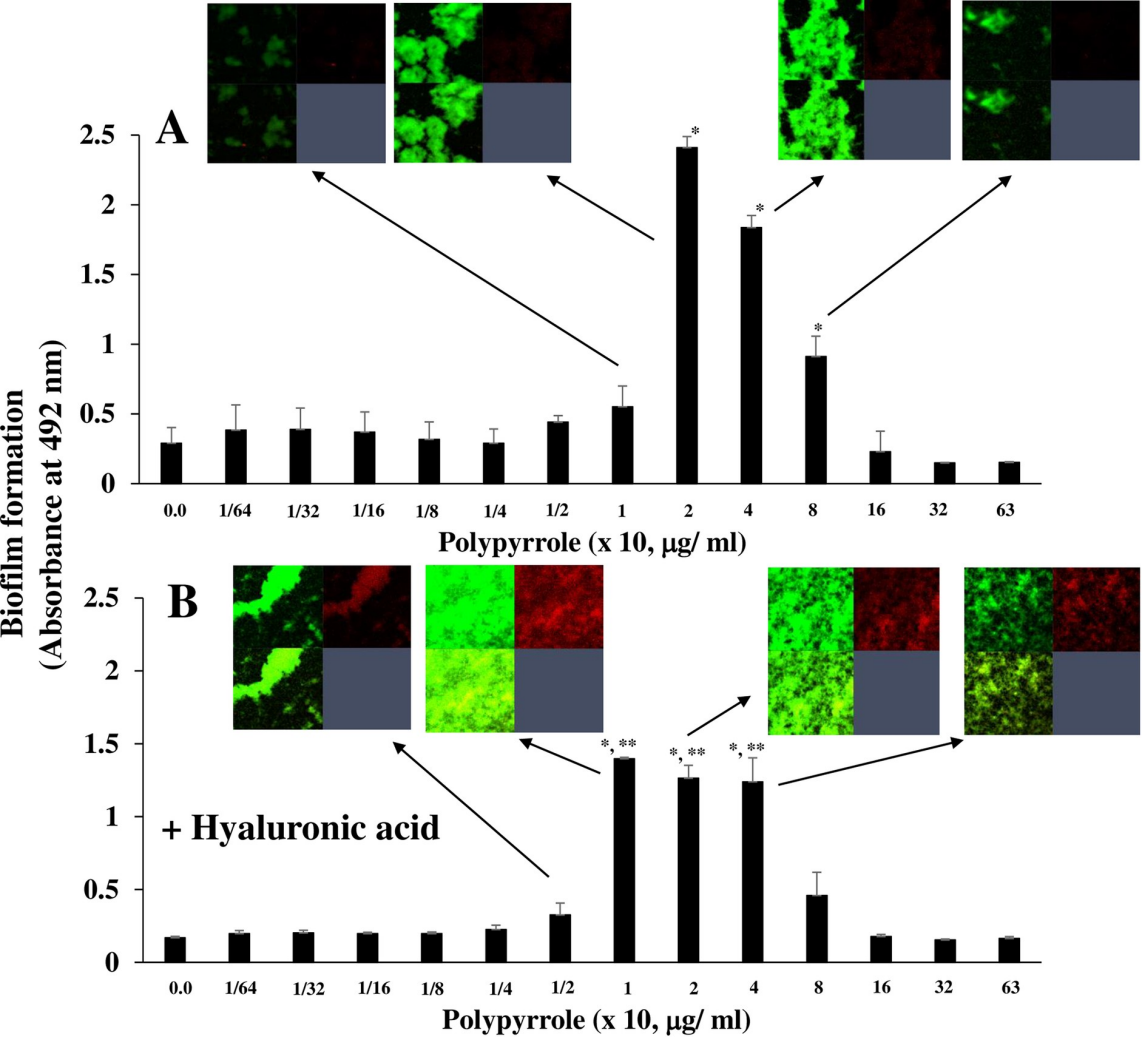
The effects of hyaluronic acid on polypyrrole-dependent biofilm formation. The effects of hyaluronic acid on the polypyrrole-dependent biofilm formation of *S*. *mutans* were observed. A: Biofilm formation by *gtfBC* mutant was quantitatively assessed in various concentrations of polypyrrole on human saliva-coated 96-well microtiter plates in TSB supplemented with 0.25% sucrose and various concentrations of polypyrrole. B: Polypyrrole was preincubated with hyaluronic acid and applied in the biofilm formation assay with *gtfBC* mutant. The data indicated the mean ± SD of triplicate experiments. The independent experiments were performed 3 times, with similar results obtained in each. The asterisks indicated a statistically significant difference among multiple groups (ANOVA with Bonferroni correction; * *p-values* < 0.05, various concentrations of polypyrrole vs no polypyrrole control: Student’s t-test; **p-values < 0.05, only polypyrrole vs polypyrrole + hyaluronic acid at 1, 2 and 4 x 10, μg/ml). Biofilms at some concentrations of polypyrrole were stained by the LIVE/DEAD BacLight Viability Kit, observed by confocal microscopy and analyzed (Zen). Confocal images for the biofilm formation are indicated by arrows and presented in the top left image (live cells), top right image (dead cells) and bottom left image (merged live and dead cells). Representative data from more than 3 independent experiments were present in the images.

To clarify the quality of the biofilm induced by polypyrrole, live and dead cells were observed by a staining kit using SYTO9 and PI. At concentrations of 20–80 μg/ml polypyrrole, live cells were largely observed in the biofilm formation as compared with dead cells (Fig 7A). To counteract the effects of positively charged proteins on the biofilm formation of *gtfBC* mutants, negatively charged hyaluronic acid (in excess over polypyrrole) was used and added to pretreat the polypyrrole in the biofilm formation assay that used various concentrations of polypyrrole. Hyaluronic acid is an anionic polysaccharide that has excellent hydrophilicity and biocompatibility [43]. Pretreating polypyrrole with hyaluronic acid, inhibited the biofilm formation of the *gtfBC* mutant at polypyrrole concentrations of 20 and 40 μg/ml (Fig 7B). However, hyaluronic acid induced dead and live cell-dominant biofilm formation at concentrations of 10–40 μg/ml polypyrrole. These dead and live cell-dominant biofilms were not easily removed by washing with DW compared with the live cell-dominant biofilms in the non-treatment with hyaluronic acid. At 10 μg/ml polypyrrole, the biofilm formation level was higher with the polypyrrole pretreated with hyaluronic acid than with the polypyrrole without hyaluronic acid. However, treatments of only hyaluronic acid could not induce biofilm formation at any concentration (S5 Fig).

## Discussion

High concentrations of polypyrrole (more than 630 μg/ml) inhibited the biofilm formation of the *S*. *mutans* laboratory strains UA159, MT8148, GS5 and clinical isolates FSC-7 and FSC-8 in conditions with insoluble glucan and increased the chaining of *S*. *mutans* UA159 in the visual analysis. The increased *S*. *mutans* chaining aggregated in natural growth and was easily removed from the solid surface by washing with DW. Polypyrrole reduced the microbial load by enhancing *S*. *mutans* cell chaining and aggregation. In previous reports, the *S*. *mutans* UA159 Δ*brpA*, Δ*atlA* and Δ*htrA* strains showed long-chain phenotypes [44–46]. The abnormal chaining in streptococci resulted in defective biofilms [44–47]. The *lytB* mutant, which encodes a putative glucosaminidase, was impaired in cell separation and showed a long-chain phenotype, resulting in impaired biofilm formation in *S*. *gordonii* [48]. Therefore, the increased chaining by polypyrrole was a negative factor for biofilm formation on the surface. Polypyrrole largely attached to the cell surface, fastened the chain of *S*. *mutans* and inhibited dispersion at high concentrations. The strong positive charge of alkaline virulence factors and ribosomal proteins most likely mediated electrostatic interactions with anionic cell surface components and anionic metabolites, as well as DNA [25]. In this study, polypyrrole showed electrostatic interactions with *S*. *mutans* cells and absorbed various proteins, including GTF-I and GTF-SI, which were produced by *S*. *mutans* during culture in the condition with sucrose. The amino acid sequence responsible for sucrose binding was proposed to be DSIRVDAVD (residues 446–454) in GTF and aspartic acid^451^ was identified as one of the active centers for catalytic activity [49, 50]. Polypyrrole might bind to the negatively charged amino acid, aspartic acid in the active site of GTFs and act by blocking the sucrose binding of GTFs. Therefore, high concentrations of polypyrrole absorbed GTF-I and GTF-SI, led to inactive GTFs, inhibited cell separation, form long chains and aggregation, and led to the physical removal from the solid surface by washing.

Polypyrrole induced biofilm formation by *gtfBC* mutants, which lacked the ability to produce insoluble glucan, at physiologically relevant concentrations in conditions with sucrose. This biofilm formation was dependent on the specific binding between bacterial cells and components coated on the plate surface, as antibodies to PAc and GbpC, which were expressed on the cell surfaces of *S*. *mutans*. The biofilm formation of initial colonizers, such as *S*. *sanguinis*, *S*. *gordonii* and *S*. *mitis*, was also induced by appropriate concentrations (2.5 and 5 μg/ml) of polypyrrole. These bacteria specifically interacted with salivary components and aggregated with other bacteria, acting as a trigger for oral biofilm formation [51, 52]. The specific interaction between the bacterial cells and binding components in human saliva were required for polypyrrole-dependent biofilm formation. Most bacterial cell surfaces were negatively charged and their interaction with human salivary components might be consolidated by the static electricity of polypyrrole at certain concentrations. It was previously reported that static electricity and physical exertion affect the adhesion and aggregation of bacteria [53, 54]. This also likely explained why bacteria adhered and aggregated more in the presence of polypyrrole. However, the biofilm formation was easily removed by increased frequency of water washing. Therefore, polypyrrole weakly supported the attachment, aggregation and biofilm formation of *S*. *mutans* and initial colonizers on human saliva-coated solid surfaces.

The biofilm formation induced by polypyrrole was live cell-dominant. Dead cells are usually induced during the process of biofilm maturation and reinforce adherence, aggregation and biofilm formation. Therefore, the live cell-dominant biofilm was abnormal in long-term culture. In contrast, dead cells appeared after polypyrrole was pretreated with hyaluronic acid in the biofilms. Therefore, the special configuration of the positive charges on polypyrrole was a main factor for biofilm formation by live cells forming weak adherence on the surface. Polypyrrole released cations and incorporated anions, reducing the pH from a neutral pH [55]. The enrichment of H^+^ in a medium could lead to protonation of the NH of pyrrole rings [56, 57] and incorporation of anions into a polypyrrole matrix. The mass increased after acid treatment and was greater than those shown in neutral pH. Polypyrrole bound cell surfaces and, at low pH conditions, might incorporate anions with organic acids, such as acetic acid, lactic acid, and others after fermentation from *S*. *mutans* during growth in TSB supplemented with 0.25% sucrose. If cations are used for the protonation of pyrrole rings, anions are incorporated by polypyrrole in these conditions. Then, stress responses will not be induced by organic acids that are produced during biofilm development because cations and anions are not sufficient for the presence of non-ionized form in acids, which consequently pass through the bacterial membrane and induce stress in the cells [58, 59]. Decreased stress can be negatively associated with the biofilm formation that occurred during stress responses, resulting in cell lysis and eDNA production [17, 18]. Moreover, polypyrrole was adsorbent for proteins such as GTF and eDNA [33, 34]. The increased mass of polypyrrole largely aggregated with *S*. *mutans* live cells and formed biofilms in appropriate concentrations. Therefore, it was considered that the live cell-dependent biofilm formation induced by polypyrrole was not reinforced by effectors such as eDNA, dead cells, and glucans and was physically and easily removed by increased frequency of water washing.

In the condition counteracted by hyaluronic acid to polypyrrole, the hyaluronic acid, which is an anionic polysaccharide, acted as a dopant for the polypyrrole [60]. Hyaluronic acid had no ability to induce biofilm formation of *S*. *mutans*. Polypyrrole films doped with low molecule hyaluronic acid were supportive of cell adhesion and growth, while polypyrrole films doped with high molecule hyaluronic acid were resistant to cell attachment in a wide range of molecular weights (3.5 x 10^3^ Da–3 x 10^6^ Da) [61]. In this assay, a wide range (3.0 x 10^3^ Da–1.5 x 10^5^ Da) of molecular weights of hyaluronic acid was used. Therefore, polypyrrole doped with hyaluronic acid played a mixed function in both the enhancement and diminishment of biofilm formation and did not incorporate cations and anions in acidic conditions after fermentation during cell growth because poplypyrrole incorporated hyaluronic acid prior to involvement of cations and anions. This indicated that treatment of polypyrrole with hyaluronic acid induces non-ionized form in low pH conditions without losing cations and anions after fermentation. The biofilm formation induced by stress responses in cells passed through the non-ionized form was reinforced by the dead cells and eDNA and hard to remove by washing in comparison with the live cell-dominant biofilm.

Biopolymers, including polysaccharides, proteins or DNA, could influence the structural integrity of biofilm matrices and bind to cations, affecting aggregation in a variety of bacterial strains [62]. Polypyrrole affected the central components of biofilm cells by promoting a structure with repeated and positive charge-regulated ionic cross-bridging and subsequent aggregation. This structure physically inhibited biofilm formation of the initial colonizers and *S*. *mutans*. Regardless of whether biofilms were formed by polypyrrole under the different properties of bacteria, the biofilms were susceptible to being washed away because of the loss of active proteins such as GTF-I and GTF-SI, dead cells and eDNA, which supported the structure of the biofilm. In conclusion, polypyrrole might be a critical material for inhibiting biofilm formation by oral streptococci and might not have side effects, such as toxicity to oral tissues and color staining on the tooth surfaces. Polypyrrole might be stable and useful as a preventive material for the prevention of biofilm-associated oral diseases. However, *S*. *mutans* clinical isolates, FSM-5 and the *S*. *sobrinus* laboratory strain OMZ176 were not inhibited by high concentrations of polypyrrole. Therefore, polypyrrole might show varying effects depending on the different properties of streptococci species on salivary component-coated solid surfaces in biofilm formation. Future investigations about the multiple effects of polypyrrole on the chaining of streptococci and inactivation of active components including GTFs, and the interaction between the bacterial cell surface and salivary components are required to understand the difference in biofilm inhibition in different streptococcal species.

## Supporting information

S1 Fig Structural formula of polypyrrole. (PPTX)

S2 Fig Inhibiting effects of polypyrrole on the biofilm formation of *S*. *mutans* in plastic tubes. (PPTX)

S3 Fig Effects of polypyrrole on *S*. *mutans* strains growth in plastic tube. (PPTX)

S4 Fig Effects of polypyrrole on the viability of epithelial cells. (PPTX)

S5 Fig Effects of hyaluronic acid on the viability of epithelial cells. (PPTX)
