# Supplementary figures and images for: The inhibitory effects of polypyrrole on the biofilm formation of Streptococcus mutans
Source: PLoS One. 2019 Nov 27;14(11):e0225584. doi: 10.1371/journal.pone.0225584 (PMC6881011; doi:10.1371/journal.pone.0225584)

## Slide 1
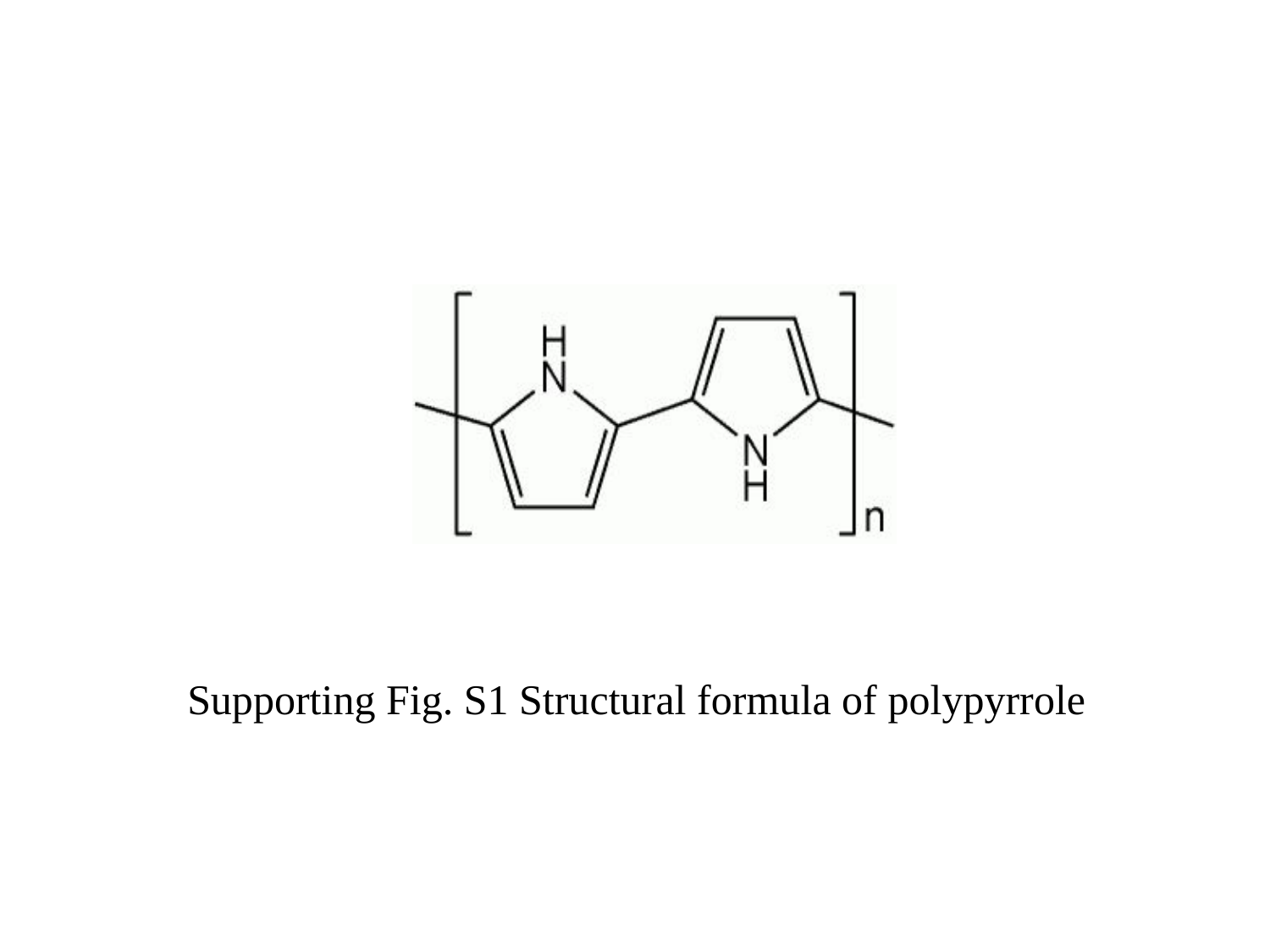

Supporting Fig. S1 Structural formula of polypyrrole

Supplement: S1 Fig — (PPTX) [file pone.0225584.s001.pptx]
