# Supplementary material for: The inhibitory effects of polypyrrole on the biofilm formation of Streptococcus mutans
Source: PLoS One. 2019 Nov 27;14(11):e0225584. doi: 10.1371/journal.pone.0225584 (PMC6881011; doi:10.1371/journal.pone.0225584)
Supplement: S2 Fig — (PPTX) [file pone.0225584.s002.pptx]

## Slide 1
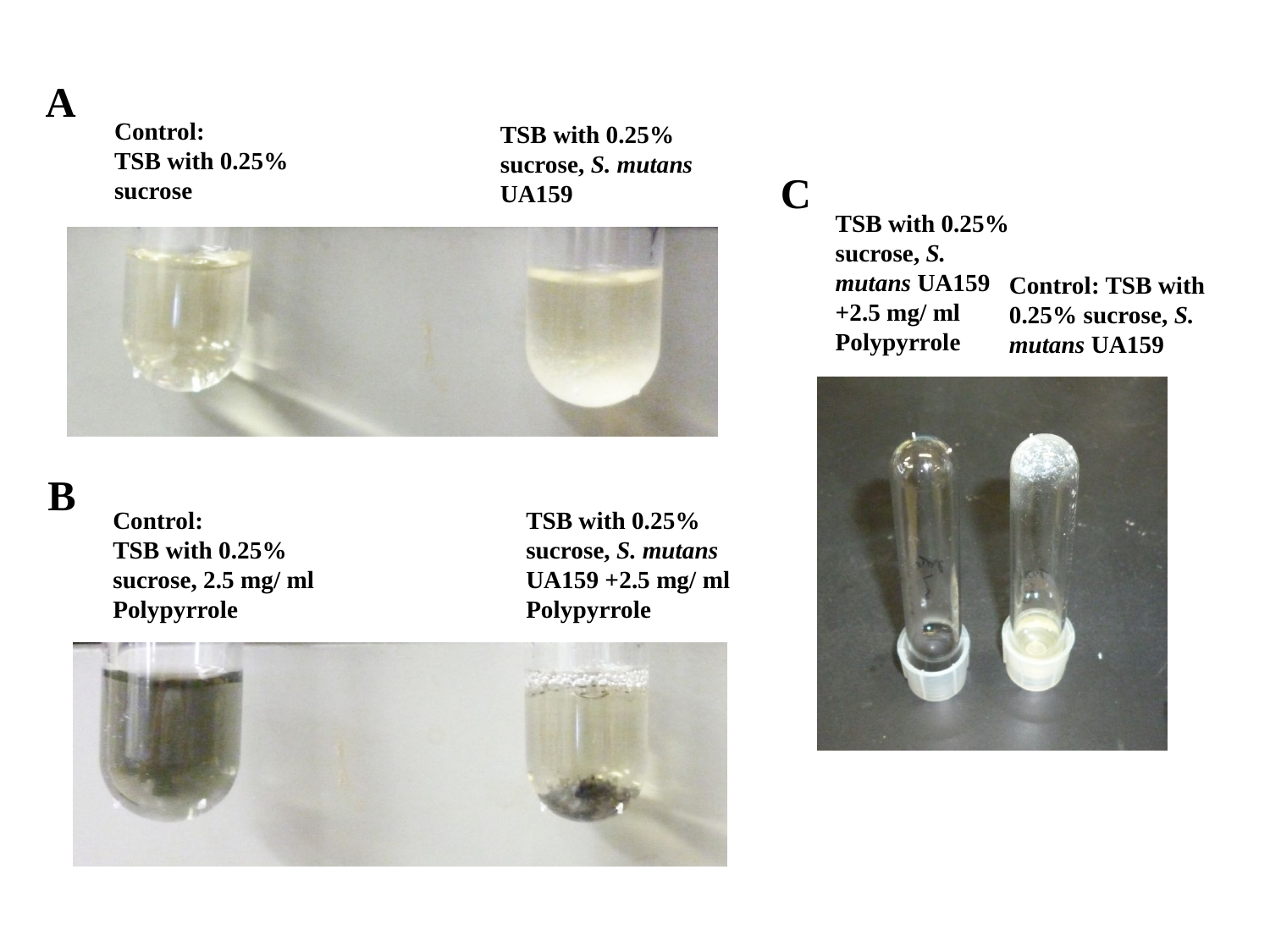

A
Control:
TSB with 0.25% sucrose
TSB with 0.25% sucrose, S. mutans UA159
C
TSB with 0.25% sucrose, S. mutans UA159 +2.5 mg/ ml
Polypyrrole
Control: TSB with 0.25% sucrose, S. mutans UA159
B
Control:
TSB with 0.25%
sucrose, 2.5 mg/ ml
Polypyrrole
TSB with 0.25% sucrose, S. mutans UA159 +2.5 mg/ ml
Polypyrrole

## Slide 2
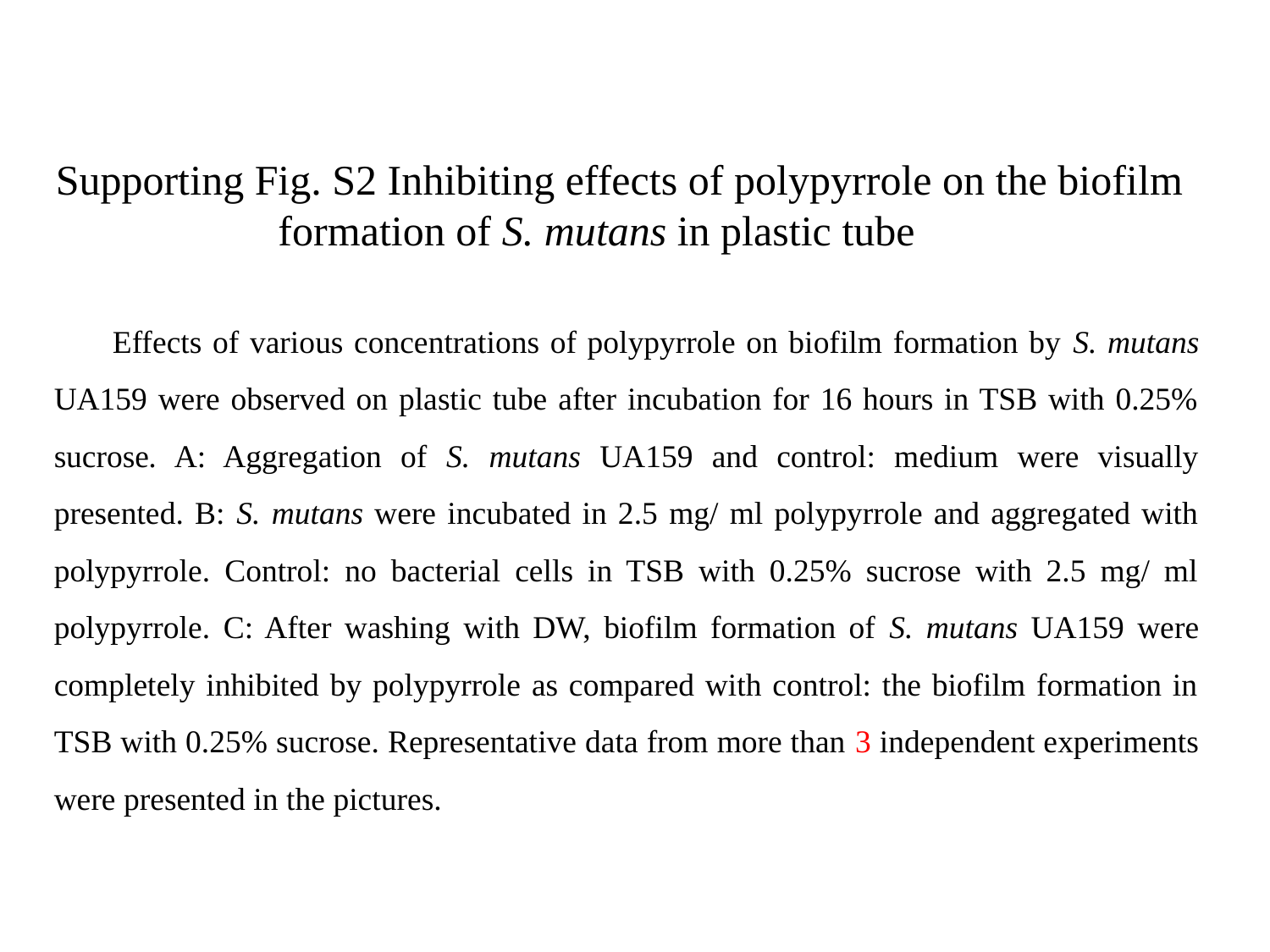

Supporting Fig. S2 Inhibiting effects of polypyrrole on the biofilm
 formation of S. mutans in plastic tube
Effects of various concentrations of polypyrrole on biofilm formation by S. mutans UA159 were observed on plastic tube after incubation for 16 hours in TSB with 0.25% sucrose. A: Aggregation of S. mutans UA159 and control: medium were visually presented. B: S. mutans were incubated in 2.5 mg/ ml polypyrrole and aggregated with polypyrrole. Control: no bacterial cells in TSB with 0.25% sucrose with 2.5 mg/ ml polypyrrole. C: After washing with DW, biofilm formation of S. mutans UA159 were completely inhibited by polypyrrole as compared with control: the biofilm formation in TSB with 0.25% sucrose. Representative data from more than 3 independent experiments were presented in the pictures.
