# Supplementary material for: The inhibitory effects of polypyrrole on the biofilm formation of Streptococcus mutans
Source: PLoS One. 2019 Nov 27;14(11):e0225584. doi: 10.1371/journal.pone.0225584 (PMC6881011; doi:10.1371/journal.pone.0225584)
Supplement: S3 Fig — (PPTX) [file pone.0225584.s003.pptx]

## Slide 1
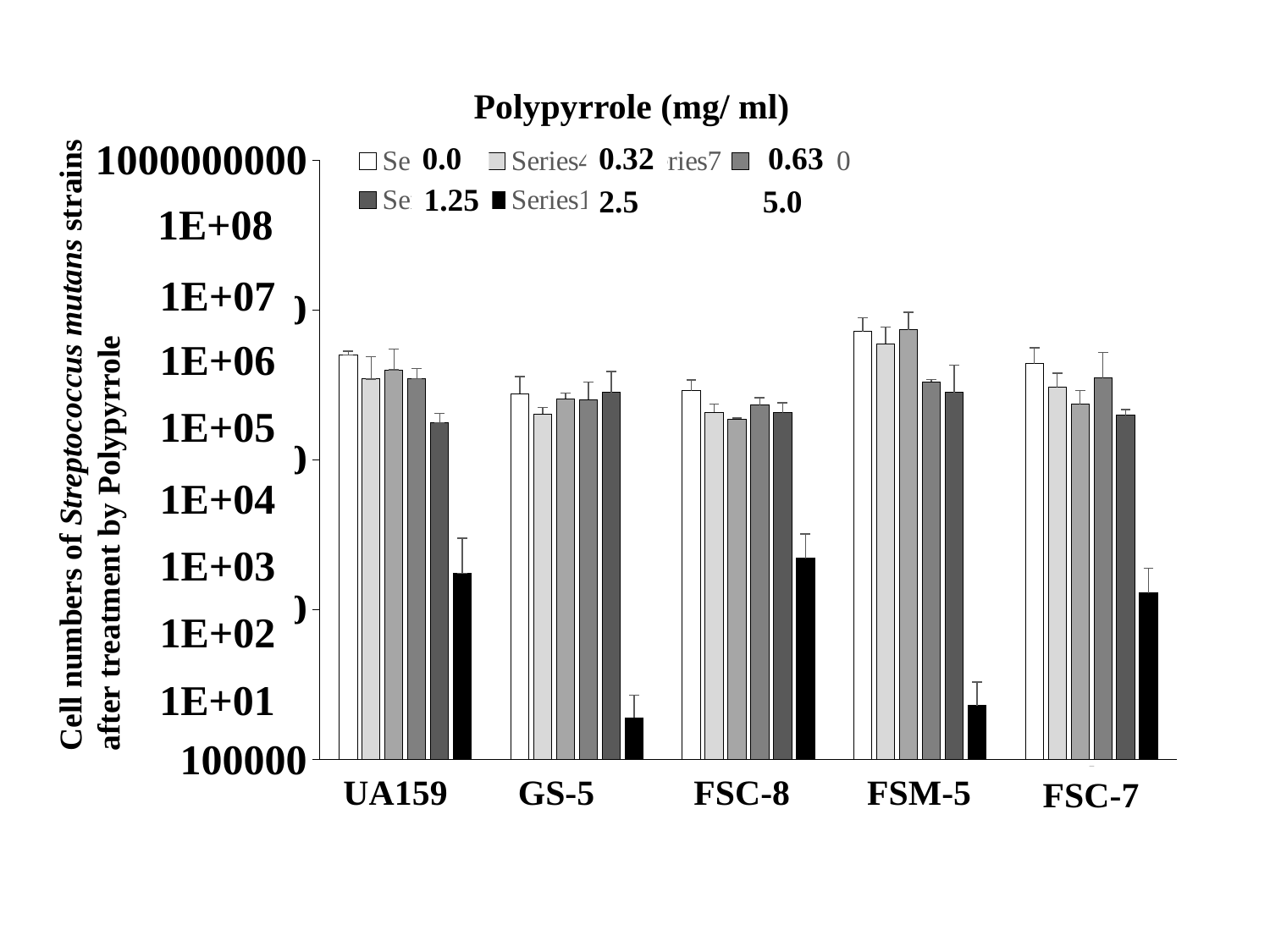

Polypyrrole (mg/ ml)
### Chart
| Category | | | | | | |
|---|---|---|---|---|---|---|0.32
0.63
0.0
1.25
2.5
5.0
1E+08
1E+07
1E+06
Cell numbers of Streptococcus mutans strains after treatment by Polypyrrole
1E+05
1E+04
1E+03
1E+02
1E+01
UA159
GS-5
FSC-8
FSM-5
FSC-7

## Slide 2
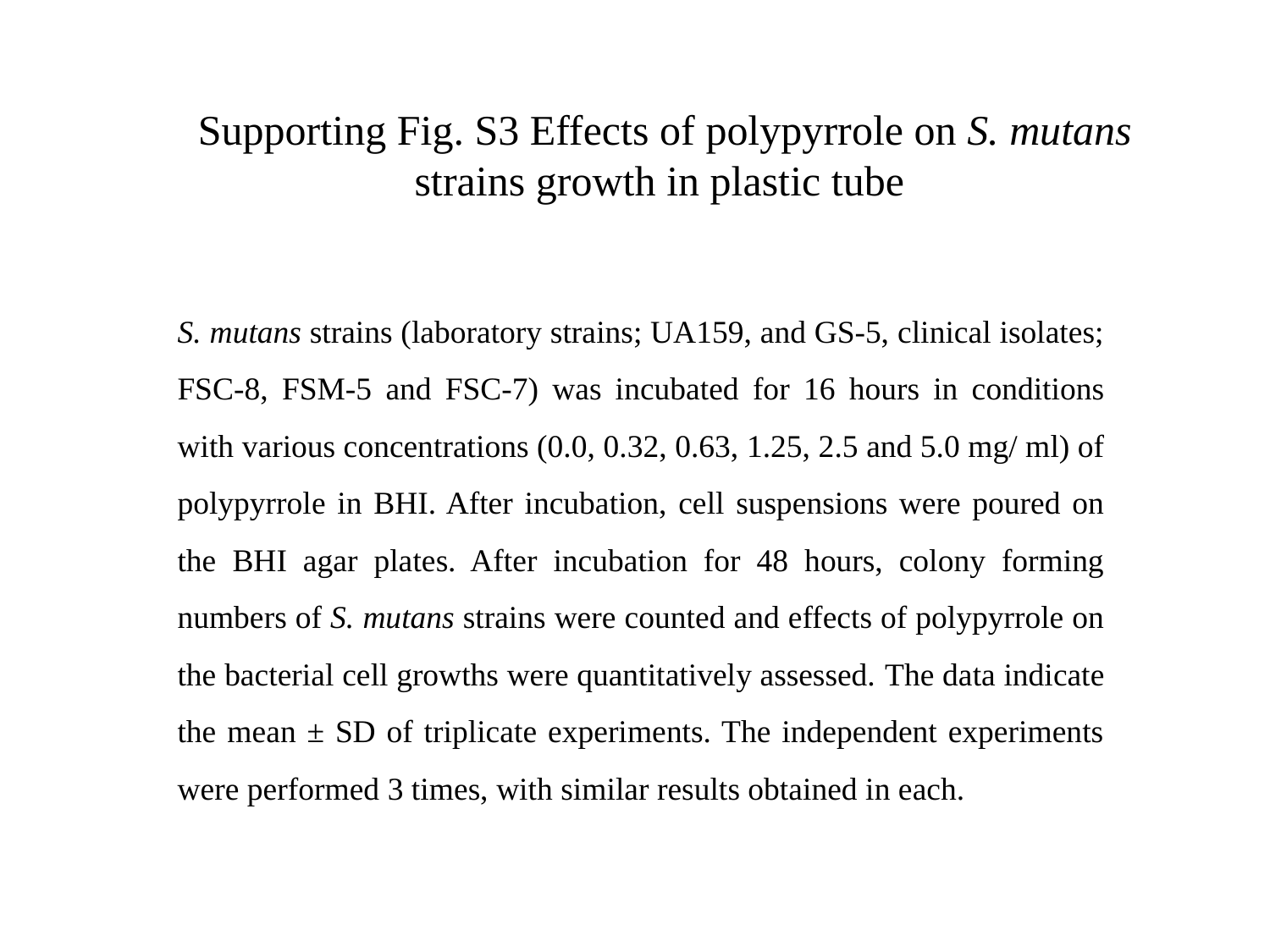

Supporting Fig. S3 Effects of polypyrrole on S. mutans strains growth in plastic tube
S. mutans strains (laboratory strains; UA159, and GS-5, clinical isolates; FSC-8, FSM-5 and FSC-7) was incubated for 16 hours in conditions with various concentrations (0.0, 0.32, 0.63, 1.25, 2.5 and 5.0 mg/ ml) of polypyrrole in BHI. After incubation, cell suspensions were poured on the BHI agar plates. After incubation for 48 hours, colony forming numbers of S. mutans strains were counted and effects of polypyrrole on the bacterial cell growths were quantitatively assessed. The data indicate the mean ± SD of triplicate experiments. The independent experiments were performed 3 times, with similar results obtained in each.
