# Supplementary material for: The inhibitory effects of polypyrrole on the biofilm formation of Streptococcus mutans
Source: PLoS One. 2019 Nov 27;14(11):e0225584. doi: 10.1371/journal.pone.0225584 (PMC6881011; doi:10.1371/journal.pone.0225584)
Supplement: S4 Fig — (PPTX) [file pone.0225584.s004.pptx]

## Slide 1
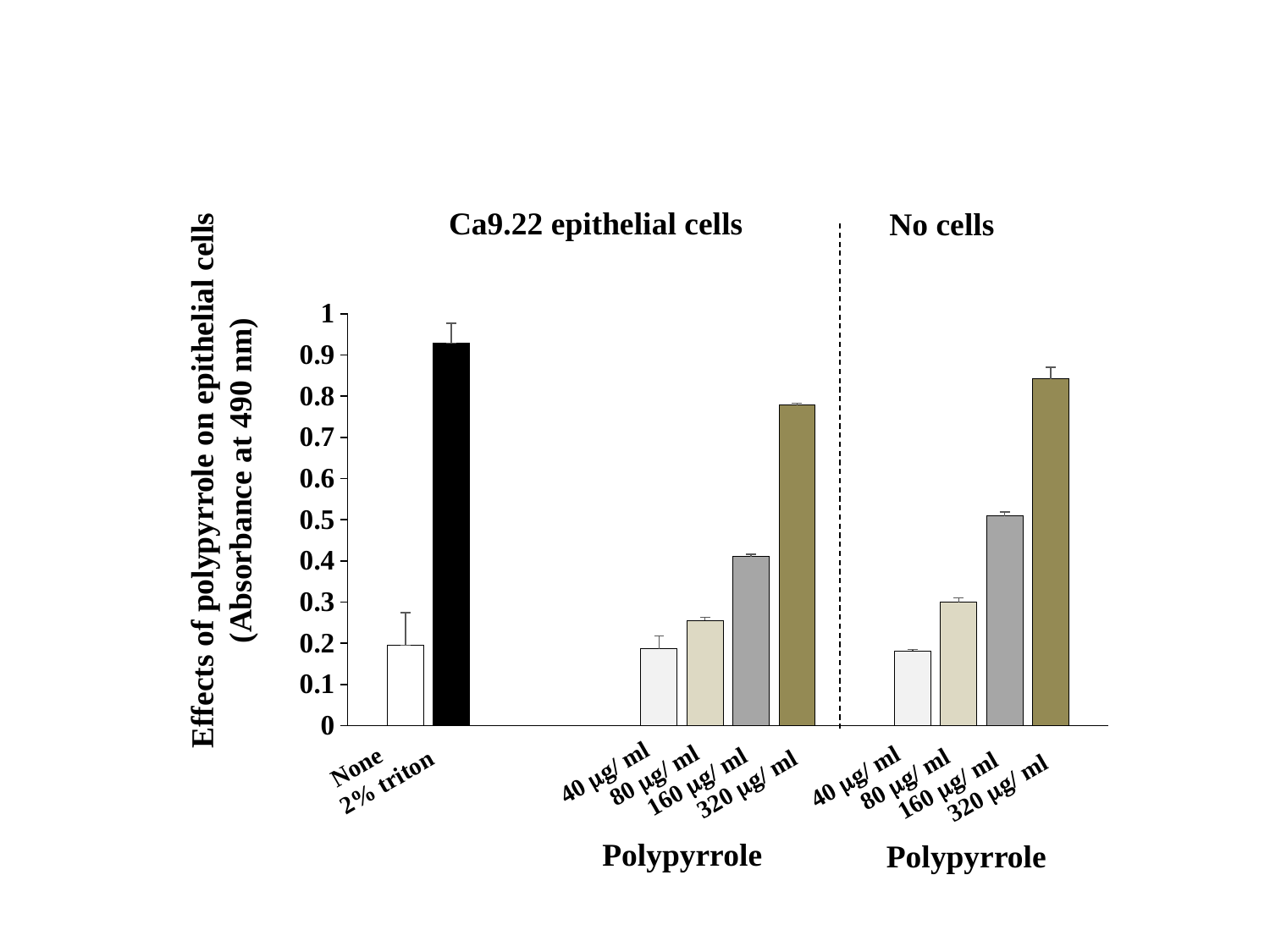

Ca9.22 epithelial cells
No cells
### Chart
| Category | | | | |
|---|---|---|---|---|Effects of polypyrrole on epithelial cells
(Absorbance at 490 nm)
None
40 mg/ ml
80 mg/ ml
40 mg/ ml
80 mg/ ml
160 mg/ ml
2% triton
320 mg/ ml
160 mg/ ml
320 mg/ ml
Polypyrrole
Polypyrrole

## Slide 2
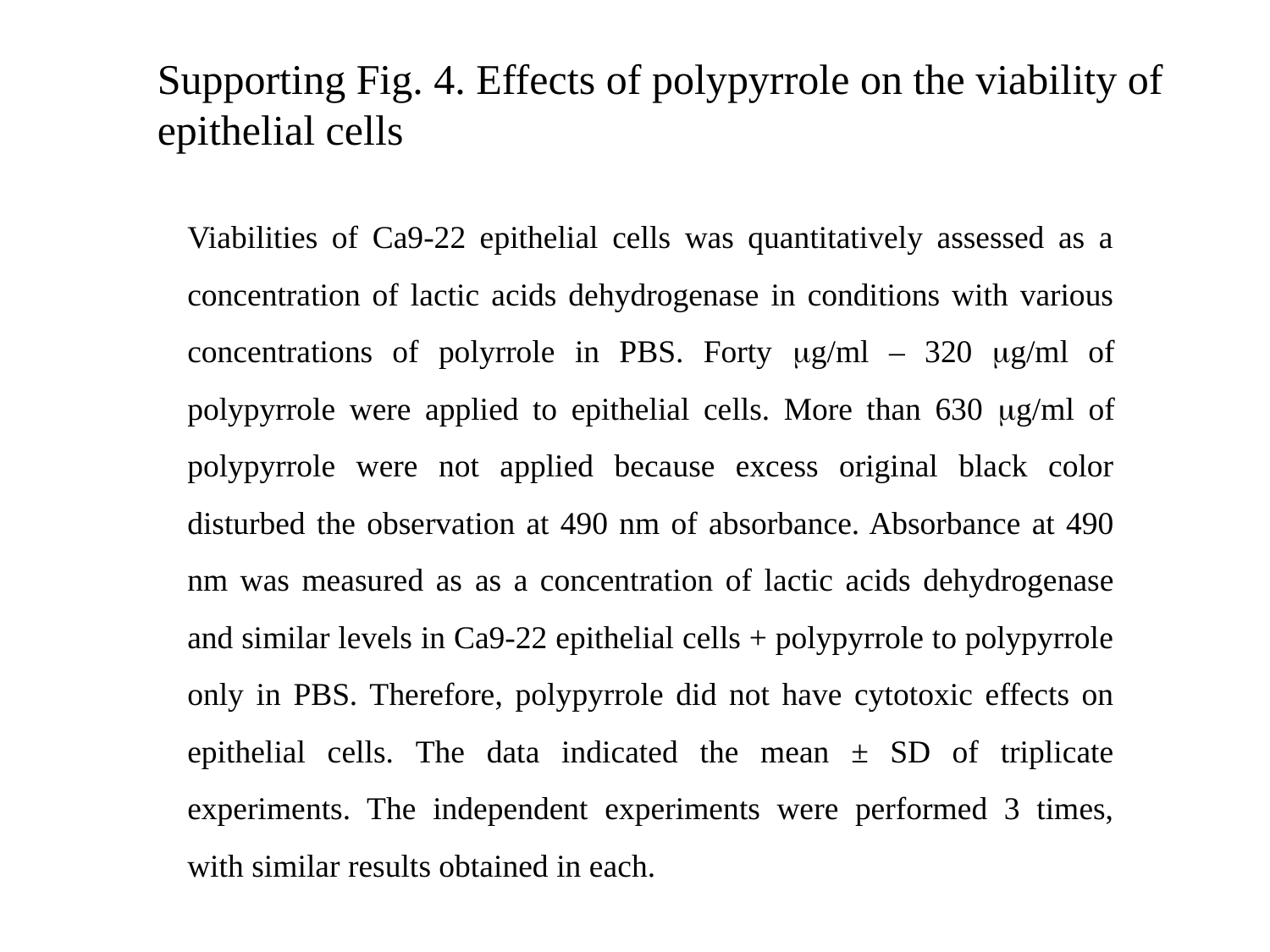

Supporting Fig. 4. Effects of polypyrrole on the viability of epithelial cells
Viabilities of Ca9-22 epithelial cells was quantitatively assessed as a concentration of lactic acids dehydrogenase in conditions with various concentrations of polyrrole in PBS. Forty mg/ml – 320 mg/ml of polypyrrole were applied to epithelial cells. More than 630 mg/ml of polypyrrole were not applied because excess original black color disturbed the observation at 490 nm of absorbance. Absorbance at 490 nm was measured as as a concentration of lactic acids dehydrogenase and similar levels in Ca9-22 epithelial cells + polypyrrole to polypyrrole only in PBS. Therefore, polypyrrole did not have cytotoxic effects on epithelial cells. The data indicated the mean ± SD of triplicate experiments. The independent experiments were performed 3 times, with similar results obtained in each.
