# Supplementary material for: The inhibitory effects of polypyrrole on the biofilm formation of Streptococcus mutans
Source: PLoS One. 2019 Nov 27;14(11):e0225584. doi: 10.1371/journal.pone.0225584 (PMC6881011; doi:10.1371/journal.pone.0225584)
Supplement: S5 Fig — (PPTX) [file pone.0225584.s005.pptx]

## Slide 1
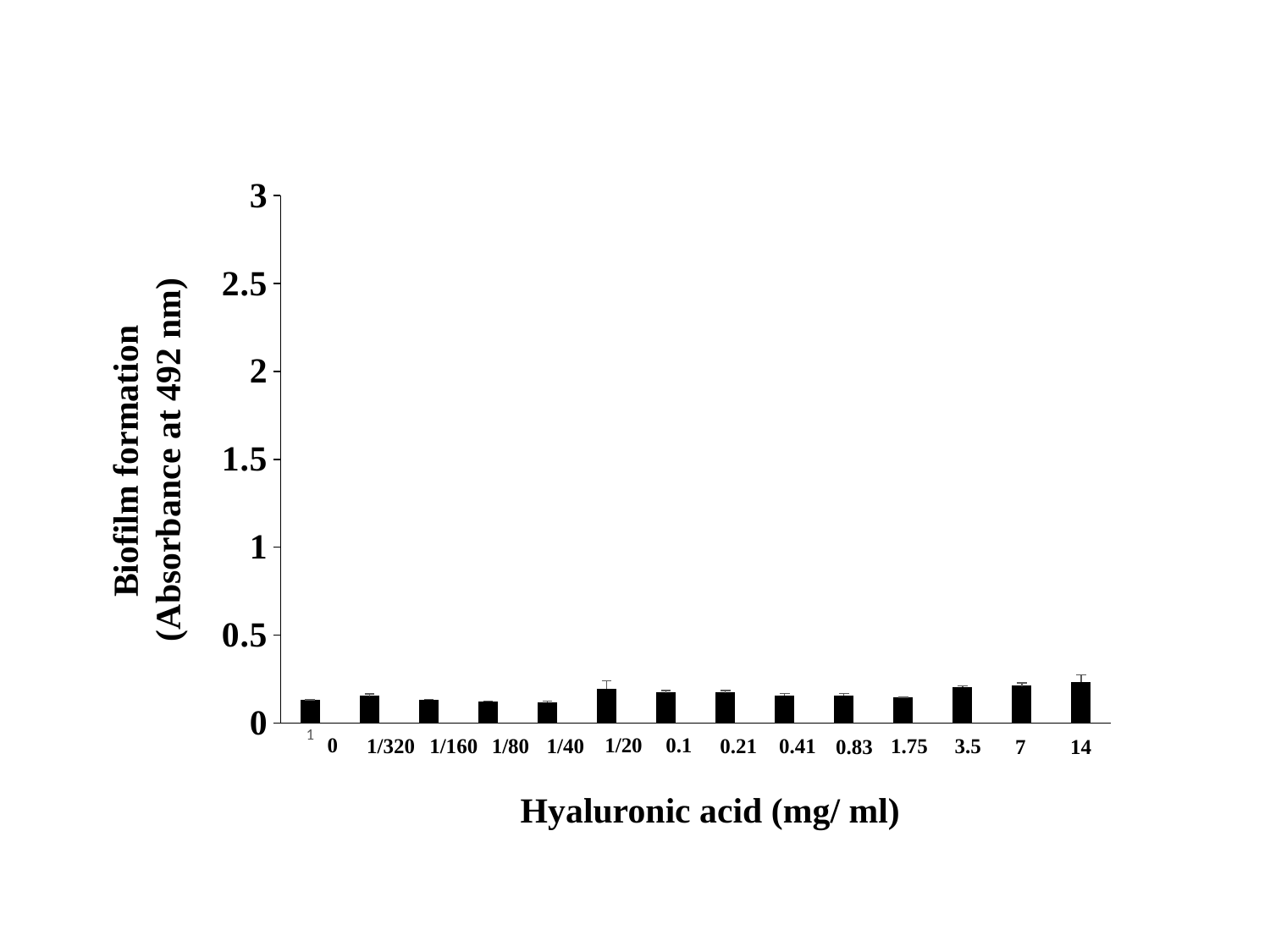

### Chart
| Category | |
|---|---| Biofilm formation
(Absorbance at 492 nm)
0
1/20
0.1
1/40
0.21
1/320
1/160
1/80
0.41
1.75
3.5
7
14
0.83
Hyaluronic acid (mg/ ml)

## Slide 2
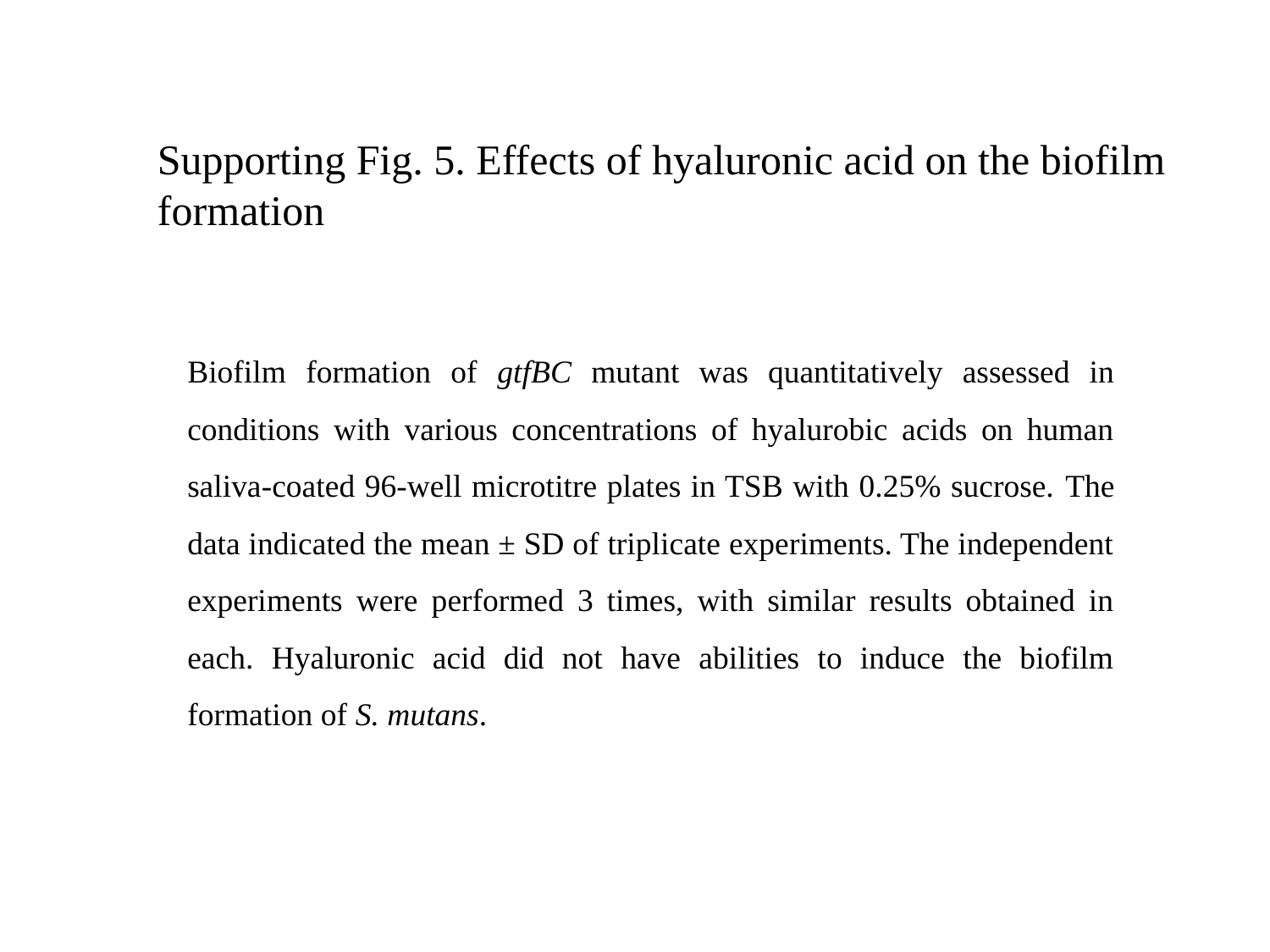

Supporting Fig. 5. Effects of hyaluronic acid on the biofilm formation
Biofilm formation of gtfBC mutant was quantitatively assessed in conditions with various concentrations of hyalurobic acids on human saliva-coated 96-well microtitre plates in TSB with 0.25% sucrose. The data indicated the mean ± SD of triplicate experiments. The independent experiments were performed 3 times, with similar results obtained in each. Hyaluronic acid did not have abilities to induce the biofilm formation of S. mutans.
